# Collagen XVII/laminin-5 activates epithelial-to-mesenchymal transition and is associated with poor prognosis in lung cancer

**DOI:** 10.18632/oncotarget.11208

**Published:** 2016-08-11

**Authors:** Chen-Chi Liu, Jiun-Han Lin, Tien-Wei Hsu, Jyuan-Wei Hsu, Jer-Wei Chang, Kelly Su, Han-Shui Hsu, Shih-Chieh Hung

**Affiliations:** ^1^ Institute of Clinical Medicine, National Yang-Ming University School of Medicine, Taipei, Taiwan; ^2^ Institute of Emergency and Critical Care Medicine, National Yang-Ming University School of Medicine, Taipei, Taiwan; ^3^ Division of Thoracic Surgery, Department of Surgery, Taipei Veterans General Hospital, Taipei, Taiwan; ^4^ Institute of Molecular and Genomic Medicine, National Health Research Institutes, Taipei, Taiwan; ^5^ Stem Cell Laboratory, Department of Medical Research, Taipei Veterans General Hospital, Taipei, Taiwan; ^6^ Institute of Biomedical Sciences, Academia Sinica, Taipei, Taiwan; ^7^ Integrative Stem Cell Center, Department of Orthopedics, China Medical University Hospital, Taichung, Taiwan; ^8^ Graduate Institute of Clinical Medical Science, China Medical University, Taichung, Taiwan

**Keywords:** Collagen XVII, laminin-5, lung cancer, cancer stem-like cells, epithelial-to-mesenchymal transition (EMT)

## Abstract

Epithelial-to-mesenchymal transition (EMT) is associated with tumor metastasis and tumorigenesis in lung cancer stem-like cells (CSCs). However, the exact mechanism underlying this is not clear. We used microarray analysis to identify candidate genes responsible for EMT in spheroid and monolayer cultures of lung cancer cells. We found increased expression of a variety of adhesion molecules in CSCs. One of these molecules, Collagen XVII (Col XVII), was demonstrated to be required for maintenance of EMT phenotypes and metastasis ability in lung CSCs. We showed that Col XVII stabilized laminin-5 to activate the FAK/AKT/GSK3β pathway, thereby suppressing Snail ubiquitination-degradation. The function of Col XVII was mainly dependent on shedding by ADAM9 and ADAM10. Patients who underwent surgical resection for lung cancer, and displayed overexpression of both Col XVII and laminin-5, had the worst prognosis of all expression types. Moreover, blockage of the Col XVII/laminin-5 pathway reduced the EMT phenotypes of lung CSCs *in vitro* and decreased the potential of lung metastasis *in vivo*. Our findings suggested that targeting Col XVII and laminin-5 could be novel therapeutic strategies for treating lung cancer patients, and warrant further investigation.

## INTRODUCTION

Lung cancer is the leading cause of cancer-related deaths in the world. The poor prognosis in lung cancer patients is associated with early relapse and metastasis following chemotherapy and radiotherapy [1]. There has been an intense focus on the study of cancer stem-like cells (CSCs) over the past decade [2–11]. CSCs comprise a specific subpopulation of tumor cells with distinct stem-like properties which allow them to initiate tumorigenesis by undergoing self-renewal and pluripotent differentiation [12]. CSCs are also known to be associated with tumor relapse and drug resistance. Dysregulation of CSC self-renewal is a likely requirement for the development of cancer. In addition, chemoresistance or survival of CSCs after treatment may be responsible for cancer recurrence or metastasis [2–11]. It has been suggested that current cancer therapies can be improved by targeting the self-renewal and survival capacities of CSCs in different kinds of cancers [13, 14]. Cancer subtypes where putative CSCs were identified, such as cancers of the brain, breast and lung, were shown to exhibit enrichment of cell populations with the properties of self-renewal and the ability to produce multi-lineage progeny in various experimental settings [2, 3, 13, 14].

Epithelial-to-mesenchymal transition (EMT) has been shown to play a critical role not only in tumor recurrence and metastasis but also in tumorigenesis [15, 16], and in the biology of CSCs [17–20]. Morel *et al*. demonstrated that mammary epithelial CD44^+^CD24^−/low^ stem-like cells could be generated from the non-tumorigenic CD44^low^CD24^+^ cells through activation of the Ras/MAPK signaling pathway. They also showed that the emergence of CD44^+^CD24^−/low^ stem-like signatures was associated with the process of EMT, as characterized by the loss of E-cadherin expression and gain of vimentin expression [17]. Ectopic expression of Twist or Snail was also shown to induce EMT in non-tumorigenic, immortalized human mammary epithelial cells, and this was accompanied by the loss of the epithelial markers and the acquisition of the mesenchymal phenotype. The cells which acquired the CD44^high^/CD24^low^ expression pattern displayed an increased mammosphere-forming ability and tumor initiating capacity [18]. Recently, Chiou *et al*. suggested that the Oct4/Nanog signaling pathway regulated epithelial-mesenchymal transdifferentiation, which promoted tumor initiation and metastasis in lung adenocarcinoma cells [19]. These reports strongly suggested that the induction of EMT generated stem-like cells; however, the molecular mechanisms responsible for such processes remain unclear.

Collagen XVII (Col XVII), a type II integral transmembrane protein, is a structural component of hemidesmosomes which connect the basal surface of epithelial cells, such as basal keratinocytes in the skin or oral mucosa, to the underlying basement membrane [21, 22]. Col XVII links the cytoplasmic structural components with the extracellular matrix (ECM) [21, 22]. The ectodomain of Col XVII is constitutively shed from the cellular surface, a process mediated by proteins of the ADAM (a disintegrin and metalloproteinase) family, mainly by ADAM9 and ADAM10. These data suggested that Col XVII plays a dual role to function as a cell surface receptor for ECM proteins, and as a matrix component [23, 24]. Most previous reports on Col XVII have focused on its role in blistering skin diseases, including bullous pemphigoid (BP) [22]. The relationship between Col XVII and lung tumorigenesis is not clear. In 1996, Yamada *et al*. reported that aberrant expression of Col XVII may reflect dysfunction of the hemidesmosome during the early event of multistep tumorigenesis of squamous epithelium, including squamous cell carcinoma of the lung [25]. In 2006, Parikka *et al*. reported that the cleaved ectodomain of Col XVII interacted with integrins to mediate the transmigration of oral squamous carcinoma cells [26]. Additional studies also suggested an association between altered expression of Col XVII and tumor transformation and progression [27–30]. The function of Col XVII and its shedding are related to keratinocyte spreading and migration, but its roles in various physio-pathological conditions are not fully understood [31].

In this study, we demonstrated that lung CSCs displayed EMT phenotypes. We used microarray analysis to identify candidate genes responsible for EMT in lung CSCs. Col XVII was among the proteins upregulated in lung CSCs. We showed that Col XVII induced EMT via stabilization of laminin-5 and upregulation of Snail expression via the FAK/AKT/GSK3b pathway. Shedding of Col XVII by ADAM9 or ADAM10 appears to be required for laminin-5 stabilization. Downregulation of Col XVII/laminin-5 or blockage of the FAK/AKT/GSK3b signaling pathway blocked the EMT phenotypes and inhibited the ability of lung CSCs to metastasize. Most importantly, we investigated the value of Col XVII and laminin-5 as prognostic markers to predict prognosis of lung cancer patients.

## RESULTS

### Lung cancer cells in spheroid culture exhibit CSC features and upregulation of EMT markers

**Figure 1:**
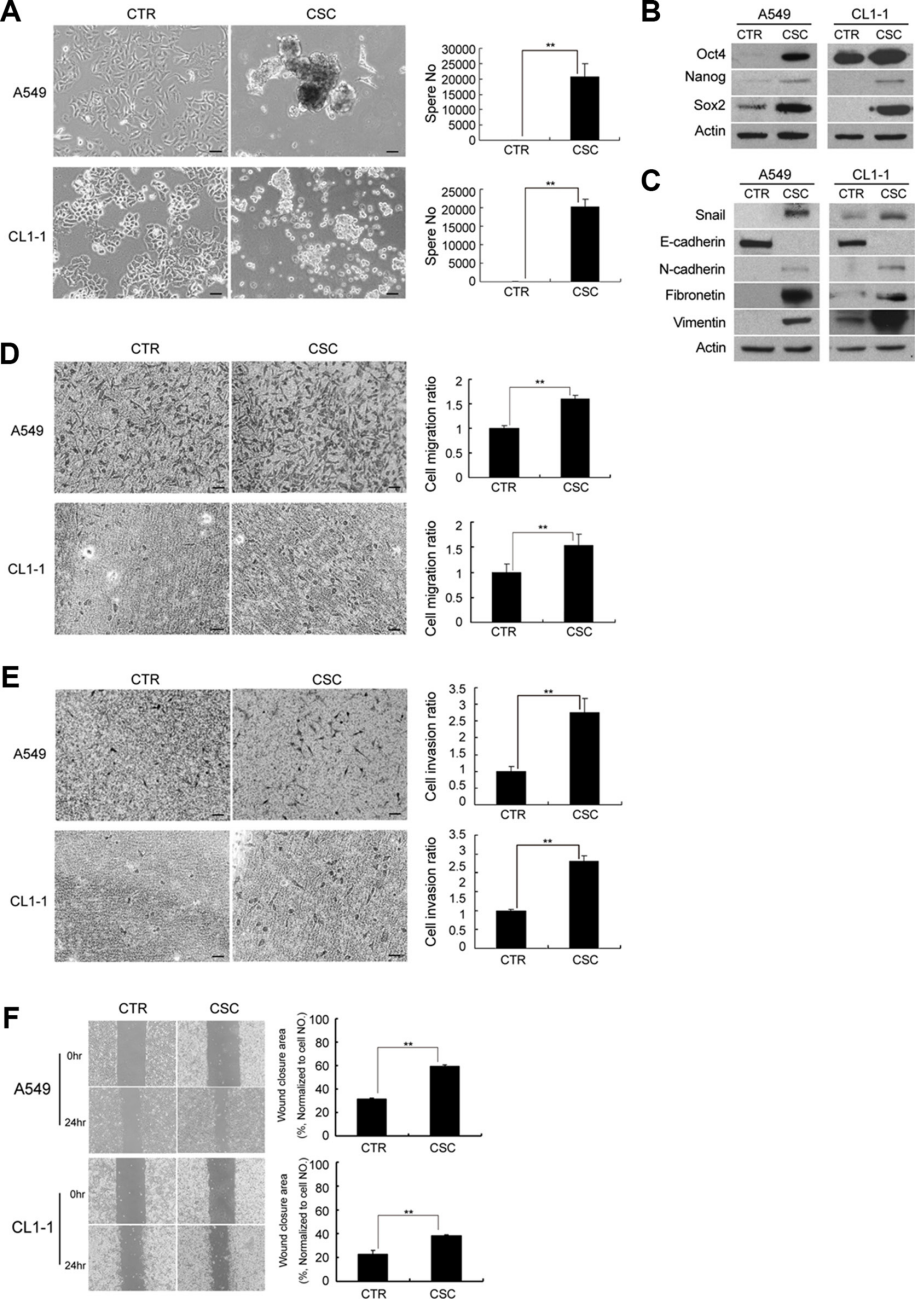
A549 and CL1-1 cells in spheroid cultures represent features of cancer stem-like cells (CSC) and epithelial-to-mesenchymal transition (EMT) **(A)** A549 and CL1-1 cells showed increased sphere formation when cultured in non-adherent culture dishes and spheroid medium for 12 days compared to cells cultured as monolayers. A549 cells cultured in adherent dishes and DMEM with 10% FBS were used as controls and no sphere formation was noted. **(B)** Western blot analysis of Oct-4, Nanog, and Sox2 in A549 and CL1-1 cells. **(C)** Western blot analysis of EMT markers, including Snail, E-cadherin, N-cadherin, fibronetin and vimentin. **(D)** Migration assay. **(E)** Invasion assay. **(F)** Wound healing assay at 0 and 24 hours. The results are expressed as mean ± standard deviation of three independent experiments. Asterisks indicate significant differences as determined by student's *t*-test or one-way ANOVA. Bars indicate 50 μm.

Lung adenocarcinoma A549 and CL1-1 cells cultured in serum-free medium supplemented with EGF and bFGF under suspension conditions initiated sphere formation at 5 days, and a continuous growth of the spheres until 12 days (Figure 1A). In contrast, A549 and CL1-1 cells cultured in DMEM supplemented with 10% FBS grew in monolayers but showed no sphere formation at 12 days. Western blot analysis demonstrated an increase in expression of the pluripotency markers, such as Oct4, Nanog, and Sox2, in spheroid culture compared to cells in monolayer culture (Figure 1B). Flow cytometric analysis (Supplementary Figure S1A) revealed that cells in spheroid culture exhibited an increase in aldehyde dehydrogenase (ALDH) activity, a hallmark of CSCs in many malignancies [8]. However, cells in spheroid culture did not show upregulation of several putative markers for lung CSCs, including CD133 and CD44 (Supplementary Figure S1B).

In addition, spheroid cultures exhibited upregulation in the expression of Snail, the transcription factor involved in EMT induction, as well as mesenchymal markers, such as N-cadherin, fibronectin and vimentin. However, the expression of epithelial markers such as E-cadherin was downregulated in spheroid cultures (Figure 1C). A549 and CL1-1 cells in spheroid culture showed increased migration (Figure 1D), invasion (Figure 1E) and wound closure ability (Figure 1F) when compared to cells in monolayers. These data suggested that lung cancer cells in spheroid culture exhibited CSC characteristics and displayed EMT phenotypes.

### Upregulation of Col XVII in lung cancer spheroid cultures is required for the maintenance of CSC characteristics and EMT phenotypes

**Figure 2:**
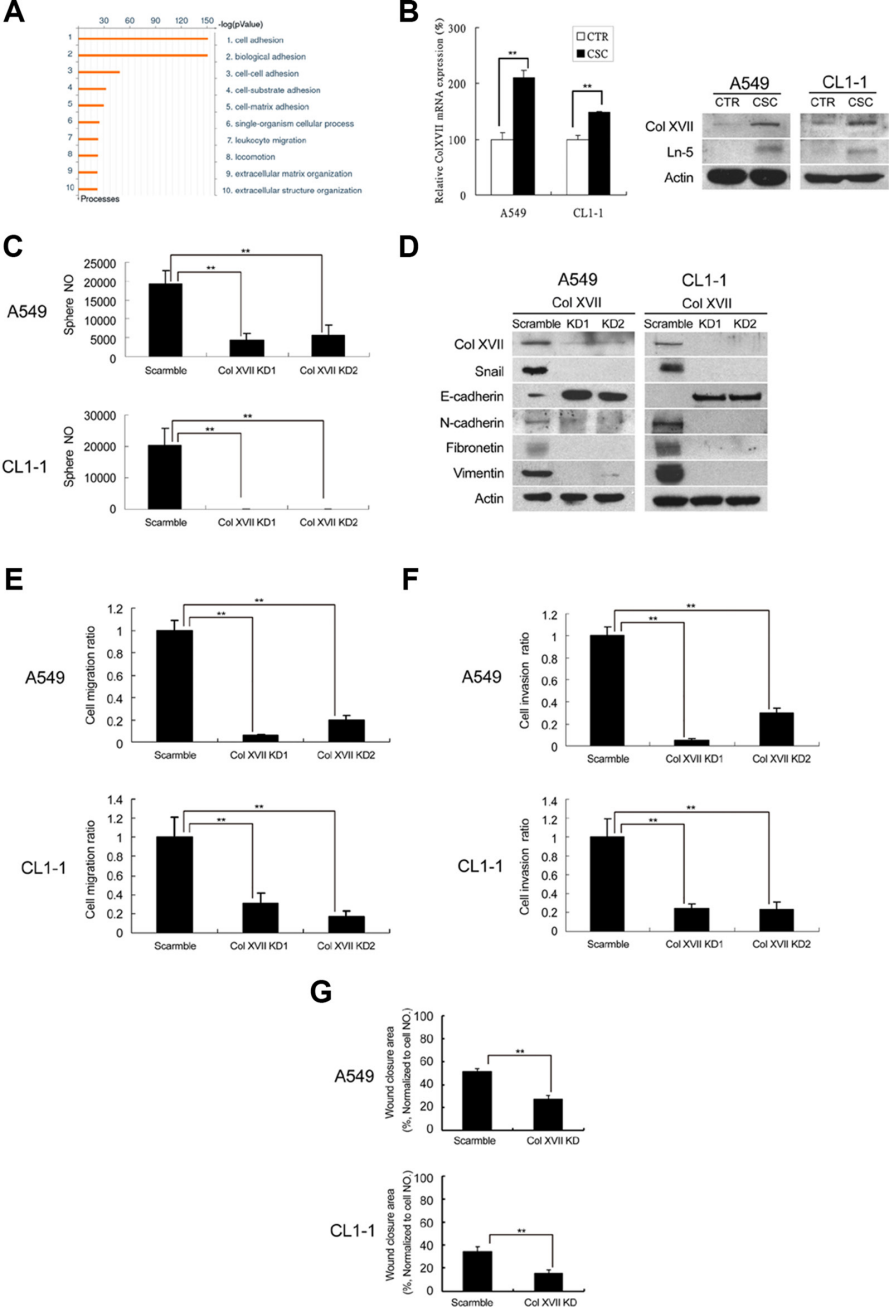
Microarray analysis showed upregulation of Col XVII and laminin-5 in lung CSCs and knockdown of Col XVII reduced the features of CSC and EMT A549 and CL1-1 cells were grown in spheroid culture (TS-LC) or adherent culture (CTR) for 12 days. **(A)** Results of microarray analysis (comparison of A549 cells cultured in serum or serum-free medium for 12 days). **(B)** Results of RT-PCR analysis of Col XVII expression in A549 cells and Western blot analysis of Col XVII and laminin-5 (Ln-5) expression in A549 and CL1-1 cells. **(C)** Knockdown (KD) of Col XVII resulted in decreased sphere formation in A549 and CL1-1 cells. **(D)** Western blot analysis showed KD of Col XVII downregulated the expression of Ln-5 and EMT markers (including Snail and N-cadherin) and upregulated E-cadherin. **(E)** Migration assay of Col XVII KD. **(F)** Invasion assay of Col XVII KD. **(G)** Wound healing assay of Col XVII KD. The results are expressed as mean ± standard deviation of three independent experiments. Asterisks indicate significant differences as determined by student's *t*-test or one-way ANOVA.

In order to identify genes responsible for CSC and EMT maintenance in spheroid culture, we performed microarray analysis to establish and compare the expression profiles of lung cancer cells in spheroid and monolayer cultures for 12 days. Gene-ontology (GO) analysis revealed that the genes differentially upregulated in spheroid cultures were mainly involved in cell adhesion, biological adhesion, cell-cell adhesion, cell-substrate adhesion, cell-matrix adhesion, single-organism cellular process and leukocyte migration (Figure 2A). Of the genes most significantly upregulated in spheroid culture (Supplementary Table S1), we chose Col XVII, a collagenous transmembrane protein and a structural component of the dermoepidermal anchoring complex [21], for further study based on its involvement in both extracellular adherence and intracellular signaling. We validated our microarray data by showing that Col XVII was upregulated at the mRNA and protein levels in spheroid cultures compared to monolayer cultures (Figure 2B). Loss-of-function approach was used to demonstrate the involvement of Col XVII in the maintenance of CSC characteristics and EMT phenotypes. Two independent shRNAs targeting Col XVII both resulted in knockdown (KD) of Col XVII, and a significant decrease in sphere formation in spheroid culture (Figure 2C). ALDH activity (Supplementary Figure S2) in spheroid culture was also suppressed by Col XVII KD compared to control shRNAs. Moreover, Col XVII KD downregulated Snail, fibronectin, vimentin and N-cadherin, but upregulated E-cadherin compared to control shRNAs (Figure 2D). Cells in spheroid culture with Col XVII KD also displayed reduced migratory activity (Figure 2E), reduced invasiveness (Figure 2F), and decreased wound closure ability (Figure 2G). Together, these data suggested that Col XVII was upregulated in lung cancer spheroid cultures and was required for the maintenance of CSC characteristics and EMT phenotypes.

### Laminin-5 works together with Collagen XVII to maintain CSC characteristics and EMT phenotypes in spheroid culture

**Figure 3:**
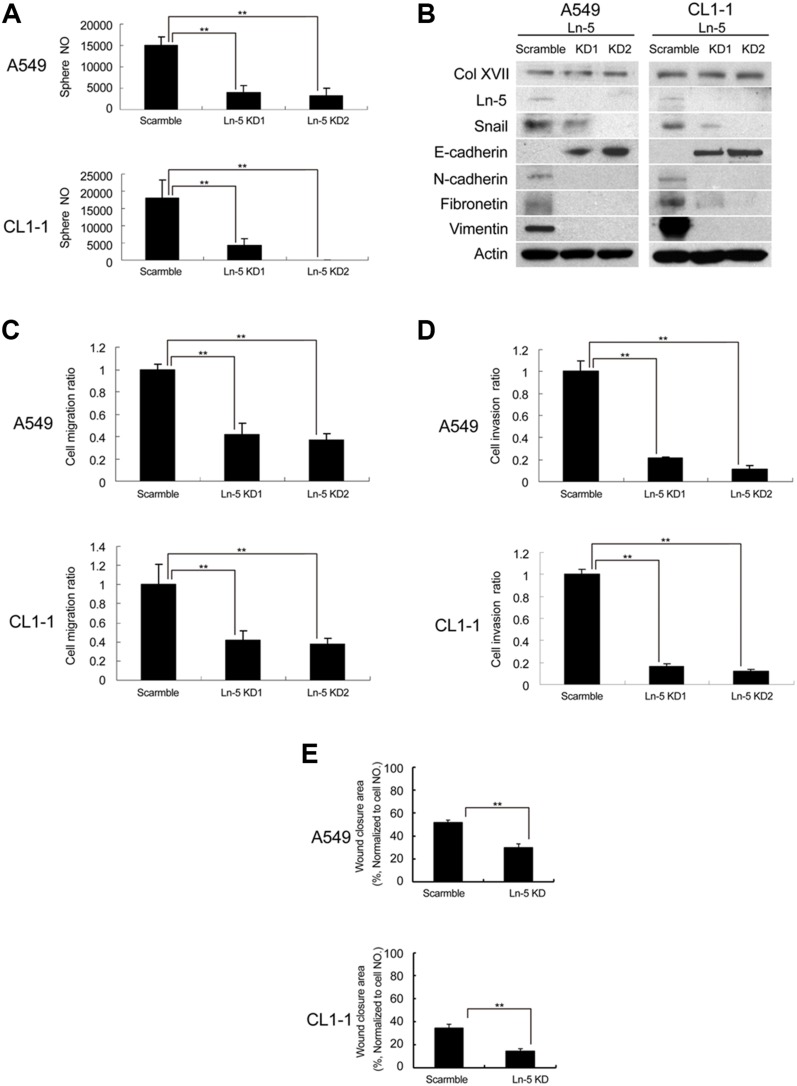
Knockdown of laminin-5 reduced the features of CSC and EMT **(A)** Knockdown (KD) of laminin-5 (Ln-5) resulted in decreased sphere formation in A549 and CL1-1 cells. **(B)** Western blot analysis showed that KD of laminin-5 downregulated the expression of EMT markers (including Snail and N-cadherin) and upregulated E-cadherin, but did not affect Col XVII. **(C)** Migration assay after Ln-5 KD. **(D)** Invasion assay after Ln-5 KD. **(E)** Wound healing assay after Ln-5 KD. The results are expressed as mean ± standard deviation of three independent experiments. Asterisks indicate significant differences as determined by student's *t*-test or one-way ANOVA.

Col XVII has been shown to interact with laminin-5 (also named laminin 332) and participate in cell/matrix interaction [32]. We examined the involvement of laminin-5 in Col XVII-regulated maintenance of EMT in CSCs. We showed upregulation of laminin-5 in spheroid cultures compared to monolayer cultures (Figure 2B). Furthermore, there was a significant suppression of sphere formation by lentiviral-mediated laminin-5 KD compared to shRNAs (Figure 3A), and this was accompanied with reduced ALDH activity (Supplementary Figure S3). Additionally, laminin-5 KD in spheroid culture suppressed the expression of Snail, N-cadherin, fibronectin and vimentin, and increased the expression of E-cadherin (Figure 3B). There was no significant difference in the expression of Col XVII between cells with and without laminin-5 KD (Figure 3B). Laminin-5 KD in spheroid cultures also reduced cell migration (Figure 3C), invasion (Figure 3D), and wound closure (Figure 3E). These data suggested that laminin-5 worked with Col XVII in supporting CSC characteristics and EMT phenotypes in spheroid cultures.

### Col XVII enhances the properties of CSCs and EMT phenotypes

**Figure 4:**
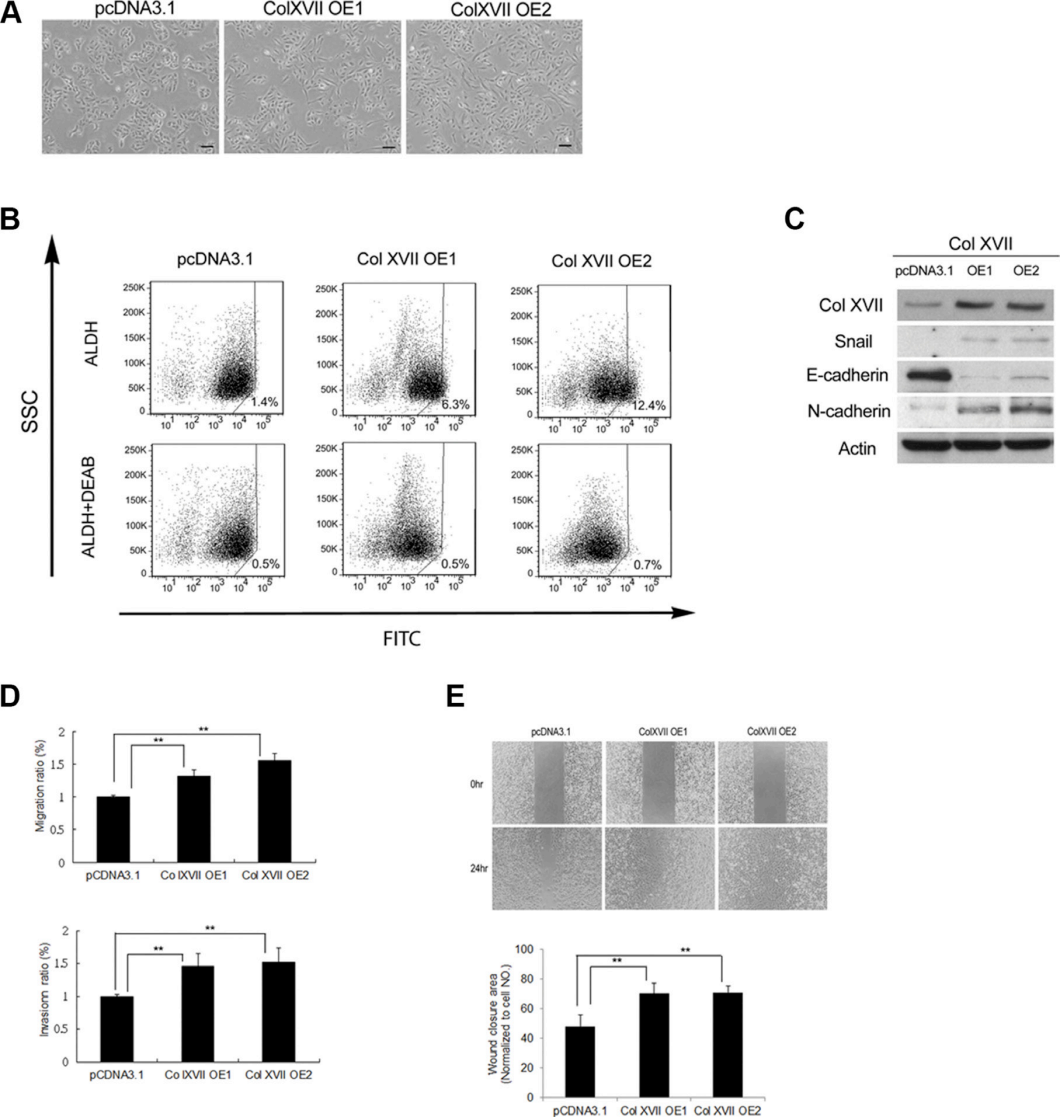
Col XVII overexpression increased CSC properties and EMT phenotypes **(A)** Overexpression (OE) of Col XVII in A549 cells cultured as monolayers resulted in spindle transformation of cell morphology compared to cells transfected with control vector pcDNA3.1. **(B)** Pictures of ALDH activity determined by flow cytometry using Aldefluor assay. **(C)** Western blot analysis showed that Col XVII OE upregulated EMT markers (including Snail and N-cadherin) and downregulated E-cadherin. **(D)** Migration and Invasion assay after Col XVII OE. **(E)** Wound healing assay after Col XVII OE. The results are expressed as mean ± standard deviation of three independent experiments. Asterisks indicate significant differences as determined by student's *t*-test or one-way ANOVA. OE1 and OE2 represent two independent single-cell clones.

Based on previous findings that Col XVII worked with laminin-5 to maintain the characteristics of CSCs and the EMT phenotypes, we overexpressed Col XVII in A549 cells and chose single cell-derived clones cultured in monolayers. We showed that cell morphology became more spindle-shaped (Figure 4A), and that Col XVII overexpression increased ALDH activity (Figure 4B) and expression of EMT markers (Figure 4C). Col XVII overexpression also increased EMT phenotypes including migration, invasion, and wound closure ability (Figure 4D and Figure 4E).

### Shedding of Collagen XVII is required to maintain laminin-5 expression and EMT phenotypes in spheroid cultures of lung cancer cells

**Figure 5:**
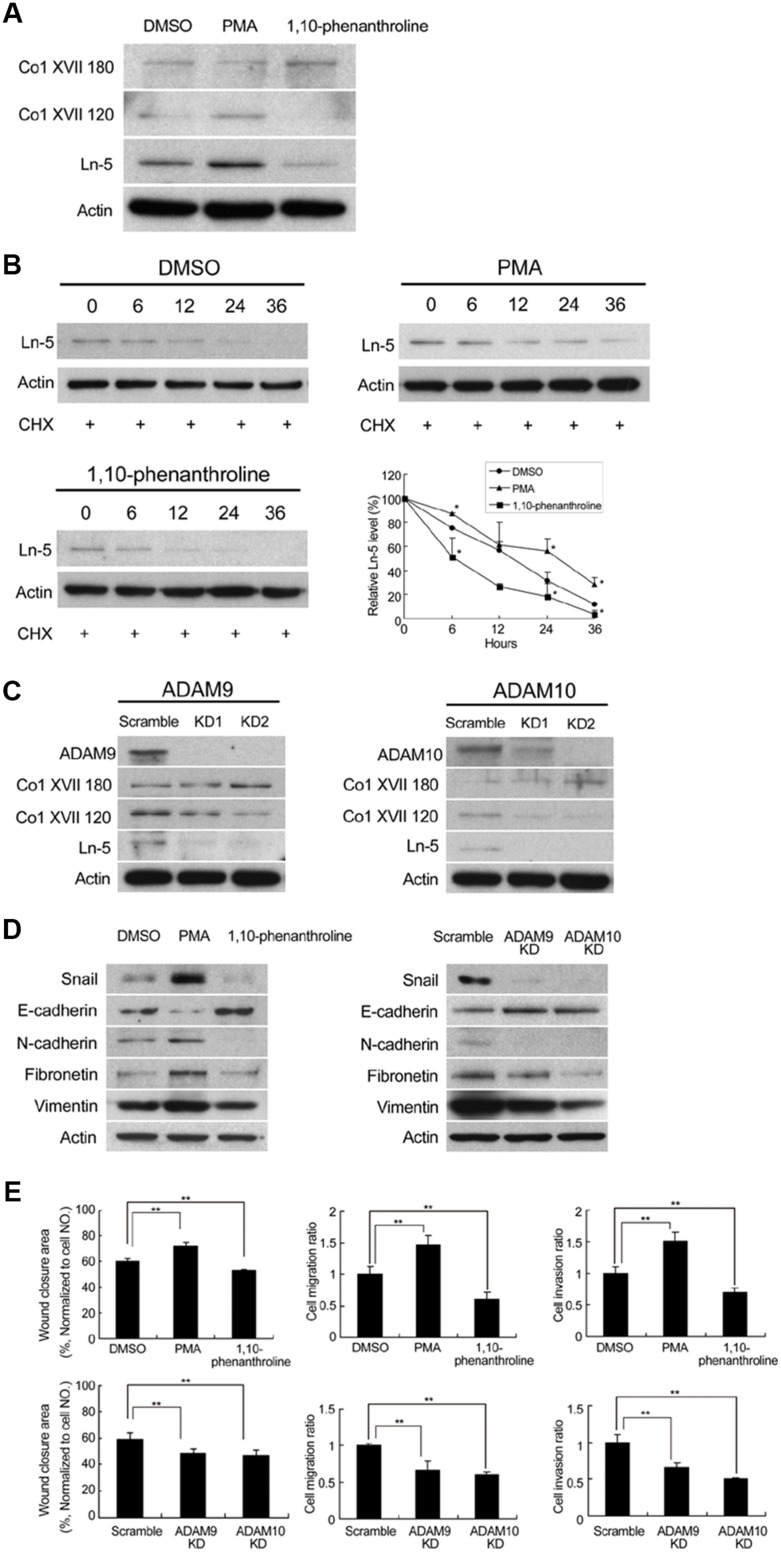
Modulation of Col XVII shedding affected laminin-5 expression. Knockdown of ADAM9 or ADAM10 inhibited shedding of Col XVII and reduced laminin-5 expression in A549 cells in spheroid culture **(A)** PMA (100 nM) or 1,10-phenanthroline (20 μM) were added to A549 cells cultured in spheroid cultured for 12 days. Cells were immunoblotted to evaluate the expression of laminin-5, the 180-kDa full-length Collagen XVII (Col XVII 180) and the 120-kDa shed ectodomain of Coll XVII (Col XVII 120, ectoderm). **(B)** Cycloheximide (50 μg/ml) was added to determine whether laminin-5 would degrade over time in A549 cells. The results of Western blot analysis showed that laminin-5 degradation was decreased by PMA and increased by 1,10-phenanthroline. **(C)** Western blot analysis showed reduced expression of Col XVII 120 and laminin-5 when ADAM9 or ADAM10 was knocked out in A549 cells. **(D)** Addition of PMA resulted in enhanced expression of Snail, and activation of the epithelial-to-mesenchymal transition (EMT) markers; addition of 1,10-phenanthroline resulted in decreased expression of Snail and EMT markers. **(E)** The results of wound healing ability, cell migration and invasion after the addition of PMA or 1,10-phenanthroline, and the same assays in ADAM9 or ADAM10 KD cells in spheroid culture.

Proteolytic cleavage of Col XVII, and its resultant ectodomain was previously shown to promote keratinocyte motility [23]. We therefore examined whether there was shedding of Col XVII in spheroid cultures of lung cancer cells. Western blot analysis showed that cells of spheroid cultures gave rise to both full-length Col XVII (180 kDa), as well as the proteolytic ectodomain fragment (120 kDa) (Figure 5A). In contrast, cells from monolayer cultures contained predominantly the full-length protein (data not shown). Incubation of suspension cells in spheroid culture medium in the presence of PMA (promoter of Col XVII shedding) or 1.10 phenathroline (inhibitor of Col XVII shedding), not only led to increased and decreased shedding of Col XVII, respectively, but also positively and negatively regulated the level of laminin-5, respectively (Figure 5A). These data suggested that Col XVII shedding was involved in the maintenance of laminin-5 protein levels, which was supported by our finding that the shedding of Col XVII did not affect the mRNA levels of laminin-5 (data not shown). However, laminin-5 protein exhibited a longer half-life in A549 suspension cells when cultured in spheroid culture medium containing cycloheximide in the presence of PMA, where shedding of Col XVII was promoted (Figure 5B). On the other hand, laminin-5 was degraded faster in cells incubated in the presence of 1.10 phenathroline, where shedding of Col XVII was suppressed (Figure 5B). The role of ADAM9 and ADAM10 in the shedding of Col XVII was investigated using a KD approach. Western blot analysis showed decreased levels of Col XVII 120 as well as laminin-5 in A549 cells in which expression of ADAM9 or ADAM10 was individually knocked down (Figure 5C). We also showed that stimulation of Col XVII shedding by PMA increased the expression of mesenchymal markers and decreased that of epithelial marker E-cadherin (Figure 5D, left panel). In parallel, inhibition of Col XVII shedding by 1,10-phenanthroline or KD of ADAM9 or ADAM10 decreased the expression of mesenchymal markers and increased that of epithelial marker E-cadherin (Figure 5D, left and right panels). Cell migration, invasion and wound closure were enhanced by PMA, and reduced by 1,10-phenanthroline, and by KD of ADAM9 or ADAM10 (Figure 5E). Together, these data suggested that laminin-5 works downstream to mediate Col XVII's effect in supporting EMT phenotype, and that ADAM9- or ADAM10-mediated shedding of Col XVII is required to stabilize laminin-5.

### Metastasis of enriched CSCs to lung depends on Col XVII and laminin-5 expression

**Figure 6:**
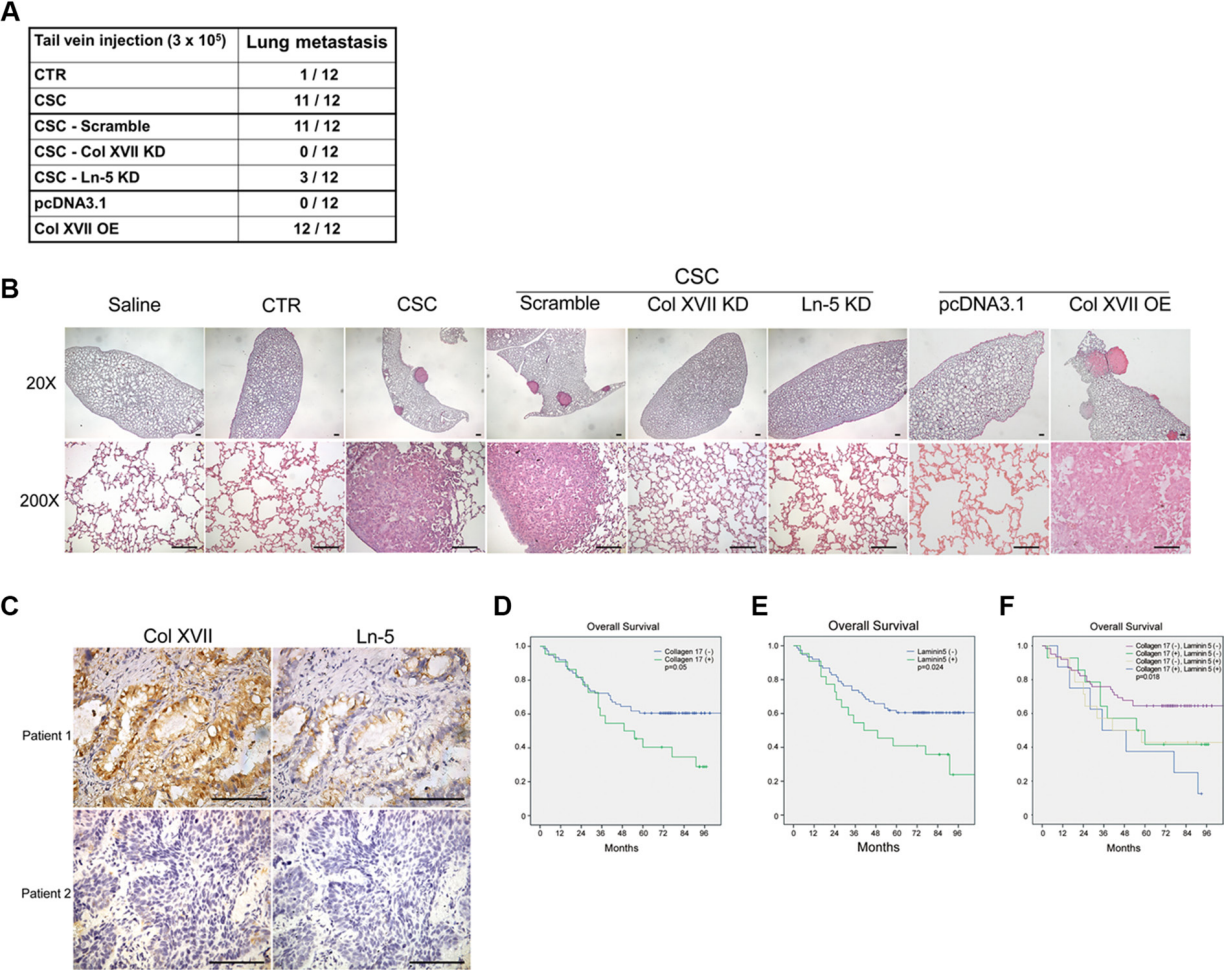
The incidence of lung metastasis was reduced by knockdown of Col XVII or laminin-5 and increased by overexpression of Col XVII in an animal model Patients with high expression of Col XVII and laminin-5 had a significantly worse prognosis after resection of lung cancer compared to patients with other expression profiles. **(A)** The results of tail vein injection of A549 cells subjected to Col XVII knockdown (KD), laminin-5 (Ln-5) KD, and Col XVII overexpression (OE). Mice injected with A549 cells cultured in spheroid cultures (CSC) showed evident lung metastasis. Control groups injected with cells cultured as monolayers (CTR), cells with Col XVII KD, and cells with Ln-5 KD in spheroid culture showed no evidence of lung metastasis at 12 weeks after tail vein injection. Col XVII OE cells cultured under monolayer conditions showed more incidence of lung metastasis when compared to cells transfected with control vector pcDNA3.1. **(B)** HE staining of lung sections showed lung metastasis in the animal receiving tail vein injection of A549 cells in spheroid culture and Col XVII OE cells cultured as monolayers. 20×, bars indicate 100 μm; 200×, bars indicate 50 μm. **(C)** Representative immunohistochemical images showing Col XVII and laminin-5 staining in tumor specimens from two patients who underwent pulmonary resection for lung cancer. Bars indicate 50 μm. **(D, E, F)** The survival curve showed that patients with positive expression of Col XVII and laminin-5 had a worse prognosis compared to patients with negative expression of Col XVII or laminin-5 or both.

To demonstrate the roles of Col XVII and laminin-5 in EMT maintenance, a process that facilitates metastasis in spheroid cultures, we performed *in vivo* metastasis assays by xenografting cells from spheroid or monolayer cultures into nude mice through tail vein injection. Lung tissues were then subjected to macro- and microscopic analyses to assess metastatic tumor formation. Inoculation of monolayer cells did not lead to lung metastasis in 12 weeks, while inoculation of the same number of spheroid cells resulted in lung metastasis in almost all mice after 12 weeks (Figure 6A). More importantly, KD of Col XVII or laminin-5 almost completely abolished the ability of the spheroid cells to form lung metastases (Figure 6A and 6B). Col XVII was overexpressed in A549 cells, and single cell-derived clones in monolayers were used to perform the lung metastasis assay. Compared to cells transfected with control vector, cells overexpressing Col XVII increased the incidence of lung metastasis (Figure 6A). These data suggested that Col XII and laminin-5 played a functional role in promoting tumor metastasis of lung CSCs *in vivo*.

### Expression of Col XVII and laminin-5 predicts poorer prognosis in patients with lung cancer

We used immunohistochemistry to investigate the clinical significance of Col XVII and laminin-5 expression in tumor specimens from a cohort of 98 patients who received lung resection for lung cancer (Figure 6C). The clinical demographics of the patients are described in Table 1. Kaplan-Meier analysis showed that patients who displayed positive staining of either Col XVII (Figure 6D) or laminin-5 (Figure 6E) in their tumors exhibited poorer survival compared to patients who were negative for both Col XVII and laminin-5 expression. Patients who displayed positive expression of both Col XVII and laminin-5 had a significantly worse prognosis than those with positive expression of either Col XVII or laminin-5 (Figure 6F). These data suggested that Col XVII and laminin-5 could be valuable markers to predict the prognosis of patients with lung cancer.

**Table 1:** Demographic data of 98 patients who underwent surgery for lung cancer

Variables (*n* = 98)	Number (%)
Age (year)	38–91 (65.18 ± 11.42)
Sex	
Male	65 (66.3)
Female	33 (33.7)
Histological type	
Adenocarcinoma	65 (66.3)
Squamous cell carcinoma	23 (23.5)
Large cell carcinoma	4 (4.1)
Mucoepidermoid carcinoma	4 (4.1)
Others	2 (2.0)
T status	
T1	13 (13.3)
T2	70 (71.4)
T3	3 (3.1)
T4	12 (12.2)
N status	
N0	58 (59.2)
N1	11 (11.2)
N2	29 (29.6)
M status	
M0	93 (94.9)
M1	5 (5.1)
Collagen XVII expression	
Increased expression (+)	22 (22.4)
Decreased expression (−)	76 (77.6)
Laminin-5 expression	
Increased expression (+)	22 (22.4)
Decreased expression (−)	76 (77.6)
Collagen XVII and laminin-5 coexpression	
Collagen XVII (+)/laminin-5(+)	8 (8.2)
Collagen XVII (+)/laminin-5(−)	14 (14.3)
Collagen XVII (−)/laminin-5(+)	14 (14.3)
Collagen XVII (−)/laminin-5(−)	62 (63.3)

### Col XVII/laminin-5 promotes EMT phenotypes in spheroid culture of lung cancer cells via the FAK/AKT/GSK3β pathway

**Figure 7:**
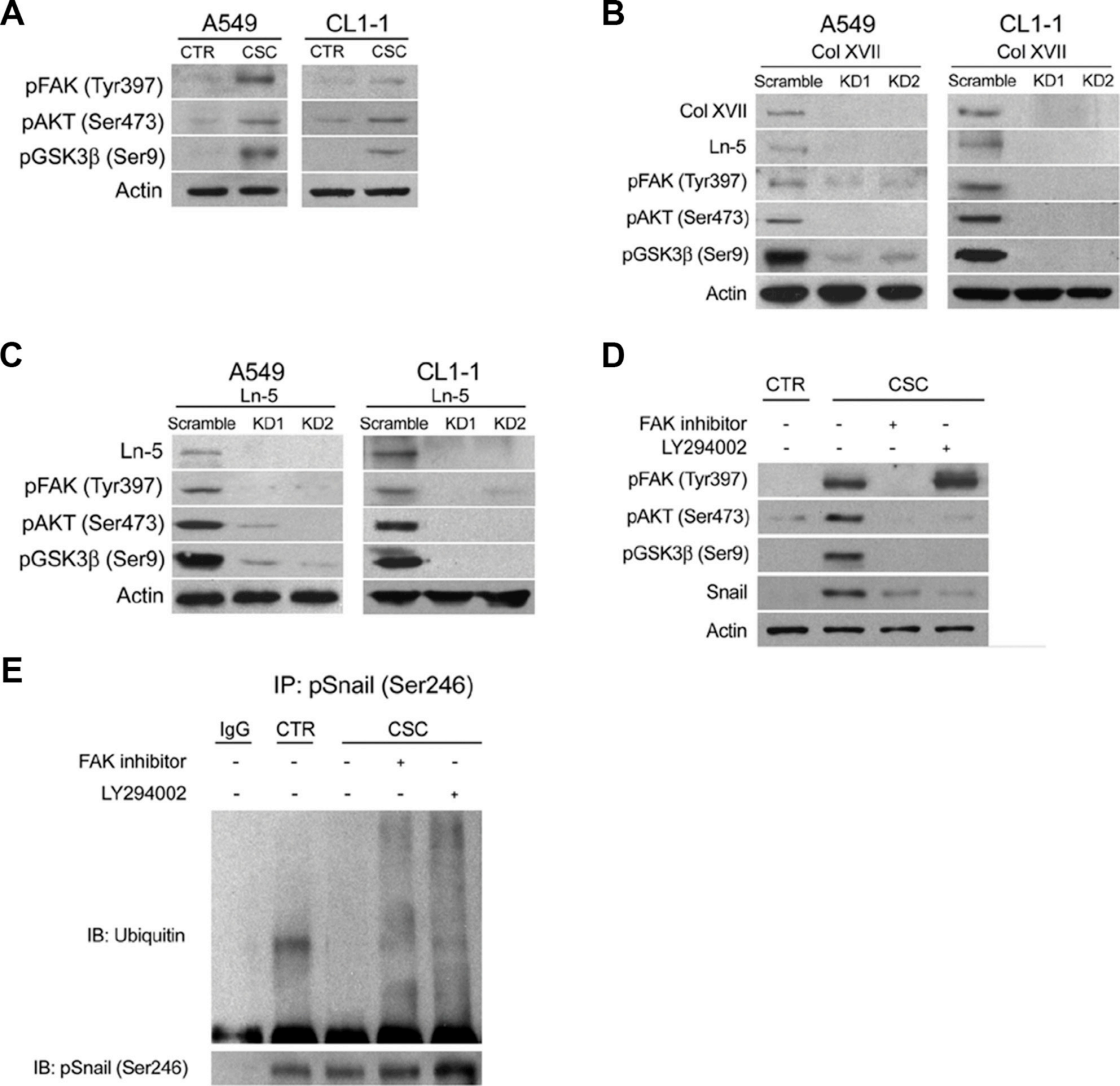
Knockdown of Col XVII or laminin-5 decreased the expression of phosphorylated FAK, AKT and GSK3β in A549 and CL1-1 lung cancer cells cultured in spheroid culture Western blot analysis of phosphorylated FAK, AKT and GSK3β expression in **(A)** A549 and CL1-1 cells cultured as monolayers (CTR) or in spheroid culture (TS-LC). **(B)** A549 cells in spheroid culture with Col XVII knockdown (KD). **(C)** A549 cells in spheroid culture with laminin-5 (Ln-5) KD and **(D)** when FAK inhibitor (20 μM) and LY 294002 (10 μM) was added, respectively. **(E**) Results of immunoprecipitation (IP) assay showed that ubiquitination of Snail was suppressed in A549 lung cancer cells cultured in spheroid culture.

We explored the mechanism(s) underlying Col XVII/laminin-5-mediated maintenance of EMT phenotypes in CSCs. Snail has been shown to be a highly labile protein which undergoes GSK3β-regulated phosphorylation and ubiquitin-mediated degradation [33]. We examined whether the AKT/GSK3β pathway was involved in the Col XVII/laminin-5-mediated upregulation of Snail expression. Western blot analysis showed increased levels of phospho-AKT and phospho-GSK3β in spheroid cultures of A549 and CL1-1 cells compared to monolayer cultures (Figure 7A). In addition, phospho-FAK levels were also significantly increased (Figure 7A). KD of either Col XVII or laminin-5 resulted in a decrease in laminin-5, phospho-FAK, phospho-AKT and phospho-GSK3β levels in A549 and CL1-1 cells in spheroid cultures (Figure 7B, 7C). When A549 cells were cultured in spheroid medium in the presence of LY294002, an inhibitor of PI3-kinase, the phosphorylation of AKT, but not the phosphorylation of FAK was blocked (Figure 7D), and this was accompanied by a decrease in phospho-GSK3β and Snail levels. Incubation of spheroid cells in the presence of an FAK inhibitor led to reduced levels of phospho-FAK, phospho-AKT, and phospho-GSK3β. These data suggested that FAK acted downstream of Col XVII/laminin-5 to induce Snail expression via the AKT/GSK3β axis. In order to support these data, we showed ubiquitination of phospho-Snail in monolayer cells, but not cells in spheroid culture (Figure 7E). Inhibitors blocking the FAK/AKT/GSK3β signaling axis facilitated Snail phosphorylation and ubiquitin-mediated degradation (Figure 7E). Together, these data suggested that activation of the FAK/AKT/GSK3β pathway was required for Col XVII/laminin-5-mediated stabilization of Snail, and for maintenance of the EMT phenotypes in spheroid cultures of lung cancer cells.

## DISCUSSION

The importance of adhesion molecules in tumor metastasis has been widely investigated. Tumor cells were reported to express a variety of adhesion molecules which mediate important cell-to-cell interactions [34, 35]. The EMT pathway is well known to be associated with tumor metastasis; however, the exact mechanism mediating activation of the EMT pathway via specific adhesion molecules is not clear [36]. We used microarray analysis to compare the gene profiles of lung cancer cell lines in serum medium or spheroid medium, and found that most genes related to cell adhesion were upregulated in cells cultured in medium supporting spheroid formation. Of these, Col XVII was activated at least 10-fold in cells cultured in spheroid medium. Further studies demonstrated that the protein levels of Col XVII and laminin-5 were increased in the lung cancer cell lines A549 and CL1-1, when cultured in spheroid medium. This study was the first to show that activation of the FAK/AKT/GSK3b pathway by Col XVII/laminin-5 suppressed ubiquitination-degradation of Snail, thereby maintaining EMT phenotypes and the metastatic ability of lung CSCs. We also demonstrated for the first time that lung cancer patients exhibiting overexpression of both Col XVII and laminin-5 had a significantly worse prognosis compared to patients overexpressing only one of these proteins, and patients negative for these proteins. Blockage of the Col XVII/laminin-5 pathway reduced the EMT phenotypes of lung CSCs *in vitro* and decreased the potential of lung metastasis *in vivo*. The 120-kDa ectodomain (Col XVII 120, ectoderm) shed by ADAM9 or ADAM10 was responsible for stabilization of laminin-5.

Col XVII, also known as BP180, is a structural component of hemidesmosomes, and mediates the adhesion of epidermal keratinocytes and certain other epithelial cells to the underlying basement membrane [21, 37]. Previous cell adhesion studies of Col XVII have mostly focused on the identification of 180-kD hemidesmosomal autoantigen in the serum of patients with BP [38, 39]. Researchers have only recently begun to investigate the role of aberrant Col XVII expression in carcinoma. Yamada *et al*. used immunohistochemistry to demonstrate enhanced expression and abnormal distribution of Col XVII in various precancerous and cancerous tissues, including Bowen's disease and squamous cell carcinoma of the cervix, lung and esophagus [25]. Parikka *et al*. found a correlation between overexpression of Col XVII and tumor progression in oral cancer [27]. However, there are no reports describing the role of Col XVII in lung tumorigenesis and metastasis. In this study, we demonstrated that activation of the Col XVII/laminin-5/FAK/AKT/GSK3b pathway enhanced the expression of Snail by reducing the level of phosphorylated Snail in lung CSCs. We also showed that the incidence of lung metastasis was reduced *in vivo* when animals were injected with lung CSCs in which Col XVII and laminin-5 expression was inhibited. These data were consistent with previous results demonstrating through a tissue microarray approach that the brain metastasis potential of non-small cell lung cancer (NSCLC) may be linked to elevated levels of Col XVII [40], and those of Fabian *et al*., who reported that increased Col XVII expression correlated significantly with brain metastasis in patients with NSCLC [41].

Col XVII is a major transmembrane glycoprotein with collagen domains in its extracellular portion [38]. Hirako et al. demonstrated the existence of a 120-kDa extracellular fragment of Col XVII which is shed from the 180-kDa molecule at the cell surface [39]. Nath *et al*. reported that shedding of Col XVII was mediated by ADAM-9 and ADAM-10, members of the ADAM family involved in cellular processes such as adhesion, migration and related signaling mechanisms [23, 24, 42, 43]. Release of the ectodomain of Col XVII from the cell surface was shown to be associated with altered keratinocyte motility and cell interactions with its environment [23]. More importantly, ADAM9 and ADAM10 expression have also been reported to be associated with various human cancers, including pancreatic cancer and lung cancer [44, 45]. Shintani *et al*. suggested that overexpression of ADAM9 enhanced the adhesion and invasion of lung cancer cells and promoted their metastasis to the brain [44]. Guo *et al*. found that ADAM10 promoted NSCLC cell migration and invasion via activation of the Notch1 signaling pathway [46]. However, the functional difference between the 180-kDa and 120-kDa fragments of Col XVII remained unclear, and the involvement of ADAM9 and ADAM10 in Col XVII shedding and in inducing EMT phenotypes in lung cancer has not been reported. To the best of our knowledge, this is the first report showing that Col XVII 180-kDa can be shed by ADAM9 and ADMA10 in the 120-kDa form, and that it subsequently influences the expression and function of laminin-5 in lung CSCs. Our data suggested that increased expression of laminin-5 activated the FAK/AKT/GSK3β pathway to stabilize Snail and induce/maintain EMT phenotypes, leading to greater cell migration/invasiveness and metastasis of lung CSCs.

Laminin-5, also known as laminin 332, is an extracellular membrane molecule involved in tumor invasion and metastasis in several different cancers [47–49]. The expression of laminin-5 in CSCs has never been reported in the literature. Microarray analysis of gene expression in lung cancer cells cultured in serum medium or spheroid medium showed a significant upregulation of cell-cell adhesion molecules, including Col XVII and laminin-5. Pyke *et al*. reported that laminin-5 might represent a valuable marker of the invasive potential of some human malignancies [47]. Giannelli *et al*. showed upregulation of laminin-5, Snail and Slug in hepatocellular carcinoma. Laminin-5 and TGF-β1 were thought to cooperatively induce EMT in hepatocellular carcinoma [48]. In this study, we investigated the relationship between activation of the Col XVII/laminin-5 pathway and EMT in lung CSCs. We found that Col XVII/laminin-5 played a role in activation of the FAK/AKT/GSK3β pathway to suppress the ubiquitination of Snail, and regulate EMT phenotypes in lung CSCs. More importantly, we also showed that overexpression of Col XVII and laminin-5 in tumor specimens was associated with poor prognosis in patients with lung cancer, and patients with overexpression of both Col XVII and laminin-5 had the worst prognosis of all expression types.

In conclusion, there is a limited understanding of the role of Col XVII and laminin-5 in lung tumorigenesis. In this study, we demonstrated the role of the Col XVII-laminin-5 pathway in the maintenance of EMT phenotypes and metastasis ability in lung CSCs via activation of the FAK/AKT/GSK3β pathway, thereby suppressing Snail ubiquitination-degradation. More importantly, lung cancer patients with overexpression of Col XVII and laminin-5 had a poor prognosis. Our findings suggested that targeting Col XVII and laminin-5 could be a novel therapeutic strategy for treatment of lung cancer patients.

## MATERIALS AND METHODS

### Cell line culture and reagents

A549 and CL1-1 lung cancer cell lines were obtained from the American Type Culture Collection. Cells were grown in Dulbecco's Modified Eagle Medium (DMEM) (Gibco, Grand Island, NY) supplemented with 10 units/ml penicillin, 10 μg/ml streptomycin, 2 mM glutamine, and 10% fetal bovine serum (FBS; Gibco), in a 37°C humidified atmosphere with 5% CO_2_. For enrichment of CSCs in spheroid culture, lung cancer cells were suspended in tumor sphere medium consisting of serum-free DMEM/F12 (Gibco), N2 supplement (Gibco), human recombinant epidermal growth factor (EGF) (20 ng/ml, PeproTech, Rocky Hill, NJ), and basic fibroblastic growth factor (bFGF) (10 ng/ml, PeproTech). Cell colonies > 100 μm in diameter and > 50% in area showing 3-dimensional structure and blurred cell margins were defined as spheres. Sphere numbers were counted on day 12 of culture. Cells were harvested and protein lysates were collected for further experiments. Treatment reagents included FAK inhibitor (20 μM) (Calbiochem, San Diego, CA; Merk, Schwalbach, Germany), LY294002 (10 μM) (Calbiochem), 1,10-phenanthroline (20 μM) (Sigma-Aldrich; St. Louis, MO), PMA (100 nM) (Sigma-Aldrich) and Cyclohexamide (50 μg/ml) (Calbiochem).

### Antibodies

Antibodies against ADAM9, phospho-AKT (Ser473), β-actin, phospho-FAK (Tyr397), ubiquitin and phospho-GSK3b (Ser9) were purchased from Cell Signaling (Boston, MA). Antibodies against laminin-5 (γ^2^ chain), Sox2, Oct-3/4, Nanog and E-cadherin were from Santa Cruz Biotechnology, Inc. (Santa Cruz, CA). Antibodies against fibronectin, vimentin and N-cadherin were purchased from GeneTex (San Antonio, TX). Antibodies against Col XVII, Col XVII (NC16A-3) and Snail were purchased from Abcam (Cambridge, MA). Antibodies against ADAM10 were from Millipore (Amersham Pharmacia Biotech, Piscataway, NJ). Antibodies against phospho-Snail (Ser246) were from OriGene Technologies, Inc. (Rockville, MD).

### Western blot analysis

Cell extracts were prepared using the M-PER protein extraction reagent (Pierce, Rockford, IL) plus protease inhibitor cocktail (HaltTM; Pierce) and protein concentrations were determined using the bicinchoninic acid (BCA) assay (Pierce). Aliquots of protein 20–40 μg lysates were separated on SDS–10% polyacrylamide gels and transferred to PVDF membrane filters. Membranes were blocked with 5% blotting grade milk (Bio-Rad, Hercules, CA) in TBST (20 mM Tris–HCl [pH 7.6], 137 mM NaCl, 1% Tween 20). Membranes were then probed with the indicated primary antibodies, reacted with the corresponding secondary antibodies, and results detected using a chemiluminescence assay (Millipore, Billerica, MA). Membranes were exposed to X-ray film to visualize the bands (Amersham Pharmacia Biotech).

### Plasmid reconstruction and cell transfection

The Col XVII overexpression plasmids were obtained from Dr. K. B. Yancey, University of Texas Southwestern Medical Center, Dallas, TX [50]. The Col XVII fragment was subcloned into pcDNA3.1 plasmids and A549 cells were transfected using TransIT-X2 Dynamic Delivery System (Mirus Bio LLC, Madison, WI, USA) according to the manufacturer's instructions. At 48 hours after transfection, cells were transferred to medium containing G418 (400 μg/ml, Sigma-Aldrich) for selection of stable clones.

### Lentiviral vector production and cell infection

The shRNA expression plasmids and bacterial clones for ADAM9 (TRCN46978, TRCN46980), ADAM10 (TRCN6674, TRCN6675), Col XVIIa1 (TRCN118937, TRCN118941) and laminin-5 (TRCN119152, TRCN119156) were provided by the RNAi Core Facility, Academia Sinica (Taipei, Taiwan). Subconfluent tumor cells were infected with lentivirus in the presence of 8 μg/ml polybrene (Sigma-Aldrich). At 24 hours post-infection, culture medium was replaced with fresh growth medium containing puromycin (4 μg/ml) to select for infected cells after 48 hours of infection.

### ALDH activity assay

A549 cells were analyzed for ALDH enzymatic activity using ALDEFLUOR assay (STEMCELL technologies, Vancouver, BC, Canada) according to the manufacturer's instructions. Briefly, 10^6^ cells were resuspended in Aldefluor assay buffer containing the ALDH substrate. As a negative control, an aliquot of Aldefluor-exposed cells was immediately treated with the specific ALDH inhibitor, diethylaminobenzaldehyde (DEBA). After 30-min incubation at 37°C, cells were analyzed using a FACSAria flow cytometer (BD Biosciences).

### Migration assay

Cell migration assay was performed using 24-well Transwell (Corning, Sigma-Aldrich). At the end of the assay, cells in the upper chamber and on the upper filter surface were removed, and cells on the lower filter surface were fixed with ethanol and stained with Giemsa (Sigma-Aldrich). The number of migrating cells was determined by counting cells in 10 random fields/filter at 200× magnification. Relative ratios to control bulk cancer cells were calculated. All experiments were performed in triplicate.

### Invasion assay

Cell invasion assays were performed using 24-well Transwell (Corning, Sigma-Aldrich) coating with Matrigel. In brief, the upper inserts were seeded with cells at a density of 2.5 × 10^4^ cells/insert in DMEM supplemented with 0.1% bovine serum albumin (BSA). Outer wells were filled with DMEM containing 10% FBS as the chemoattractant. After 24 hours of incubation, the membranes with invaded cells were stained with Giemsa (Sigma), washed and mounted on slides. The entire membrane with invading cells was counted by light microscopy. Relative ratios to control bulk cancer cells were calculated. Each assay was performed in triplicate and repeated at least twice.

### Wound healing assay

We used an *in vitro* model to measure wound healing ability by evaluating the ability of A549 and CL1-1 lung cancer cells to migrate in a monolayer culture. Lung cancer cells were seeded into 6-well plates and incubated overnight. The cells were disrupted by scraping them with a 200 μl pipette tip. Migration of cells into wounded areas of the plate was observed at 24 hours. The percent of wounded area filled in was calculated as follows: [(mean wound width-mean remaining width) / mean wound width] × 100 (%) [51]. For normalizing the interference of cell proliferation during wound healing, the percent of wound closure area was divided by the ratio of cell numbers counted at the beginning and at 24 hours after migration. All experiments were performed in triplicate.

### Microarray and data analysis

We compared the gene expression pattern after culturing A549 lung cancer cells for 12 days in a spheroid (3D) culture or in a traditional monolayer (2D) culture. Total RNA was isolated with TRIzol reagent (Invitrogen, Carlsbad, CA) according to the manufacturer's protocol. Each sample was processed and analyzed using the Affymetrix Human U133 plus 2.0 array chip (Affymetrix, Santa Clara, CA) at the National Microarray and Gene Expression Analysis Core Facility (National Research Program for Genomic Medicine, Taipei, Taiwan). Array data were analyzed using GeneSpring GX v12 software (Agilent Technologies, Santa Clara, CA), and classified using Gene Ontology terms. Microarray data were deposited in the Gene Expression Omnibus (www.ncbi.nlm.nih.gov/geo/) with an accession number of GSE80097.

### Quantitative real-time polymerase chain reaction (PCR)

Total RNA was extracted using TRIzol reagent (Invitrogen, Carlsbad, CA) and reverse-transcribed using Superscript II (Invitrogen) according to the manufacturer's instructions. The samples were analyzed with SYBR Green Master (GeneMark, Georgia Institute of Technology, Atlanta, GA) and ABI Step One Real-Time PCR System machine (Applied Biosystems, Carlsbad, CA). The specific primers used for PCR were: Col XVIIA1 (forward, 5′-AAAGGACCAATGGGACCACC-3′; reverse, 5′-TT CACCTCTTGGGCCTTGGT-3′).

### Immunoprecipitation assay

Aliquots of 500 μg cell lysate were incubated with 2 μg antibody in 500 μl IP Lysis/Wash Buffer (Pierce/Thermo Scientific), with gentle rocking overnight at 4°C, following which 25 μl Protein A/G Magnetic beads (Pierce/Thermo Scientific) were added and incubation was continued with gentle rocking for another 2 hours at 4°C. The beads were collected with a magnetic stand and the unbound sample was discarded. The precipitate was washed 2–3 times by adding 500 μl Lysis/Wash Buffer, followed by replacement with 500 μl of ultra-pure water. The beads were gently mixed and collected on a magnetic stand, followed by removal of the supernatant and dilution with 50 μl sample buffer. After adequate vortexing, the sample was denatured at 100°C for 10 minutes. The beads were magnetically separated and the supernatant was saved in a new microcentrifuge tube. Samples were immunoblotted with appropriate antibodies.

### Animal model of lung metastasis

Male Balb/C nude mice colonies were maintained in specific pathogen-free conditions. The mice were used for experiments at 7 weeks of age. Tumor cells A549 (spheroid or non-spheroid culture) without/with Collagen XVII or laminin-5 knockdown (shRNA) and A549 cells with Col XVII overexpression and control vector pcDNA3.1 (3 × 10^5^, 8 groups, 12 mice in each group) were injected in the tail vein for evaluation of lung metastasis 12 weeks later. The lung tissue of mice was embedded in paraffin and sequentially stained with H&E.

### Patients and immunohistochemistry

### Patients

Ninety-eight patients who underwent surgical resection for lung cancer in Taipei Veterans General Hospital (Taipei, Taiwan) were enrolled in this study. None of these patients received neoadjuvant chemotherapy or radiotherapy. The clinical data including the sex, age, TNM Classification of Malignant Tumors (TNM) status and disease-specific survival were reviewed and calculated. The Institutional Review Board of Taipei Veterans General Hospital approved the protocol for this study (2013-10-030CC) and patients signed the informed consents.

### Immunohistochemistry

Immunohistochemistry was used to evaluate Col XVII and laminin-5 expression in resected tumor tissues. Paraffin blocks of tumors were cut into 4-μm slices and then processed using standard deparaffinization and rehydration techniques. Anti-Col XVII antibody (1:50) and anti-laminin-5 polyclonal antibody (1:50) were used as the primary antibodies to detect the protein expression Col XVII and laminin-5, respectively. For Col XVII and laminin-5 immunostaining, positive expression was defined as detectable immunoreaction in perinuclear and other cytoplasmic regions of > 25% of the cancer cells.

### Statistical analysis

Values are shown as the mean ± standard deviation of the mean of measurements of at least three independently performed experiments to minimize variation between cell cultures. Student's *t*-test or one-way ANOVA was employed. Survival curves were calculated using the Kaplan-Meier method and comparisons were performed using the log-rank test. *P* < 0.05 was considered to be statistically significant.

## SUPPLEMENTARY MATERIALS FIGURES AND TABLE


