# Supplementary material for: Collagen XVII/laminin-5 activates epithelial-to-mesenchymal transition and is associated with poor prognosis in lung cancer
Source: Oncotarget. 2016 Aug 11;9(2):1656–72. doi: 10.18632/oncotarget.11208 (PMC5788589; doi:10.18632/oncotarget.11208)
Supplement: Supplementary file 1 [file oncotarget-09-1656-s001.pdf]

Collagen XVII/laminin-5 activates epithelial-to-mesenchymal transition and is associated with poor prognosis in lung cancer

Supplementary Materials

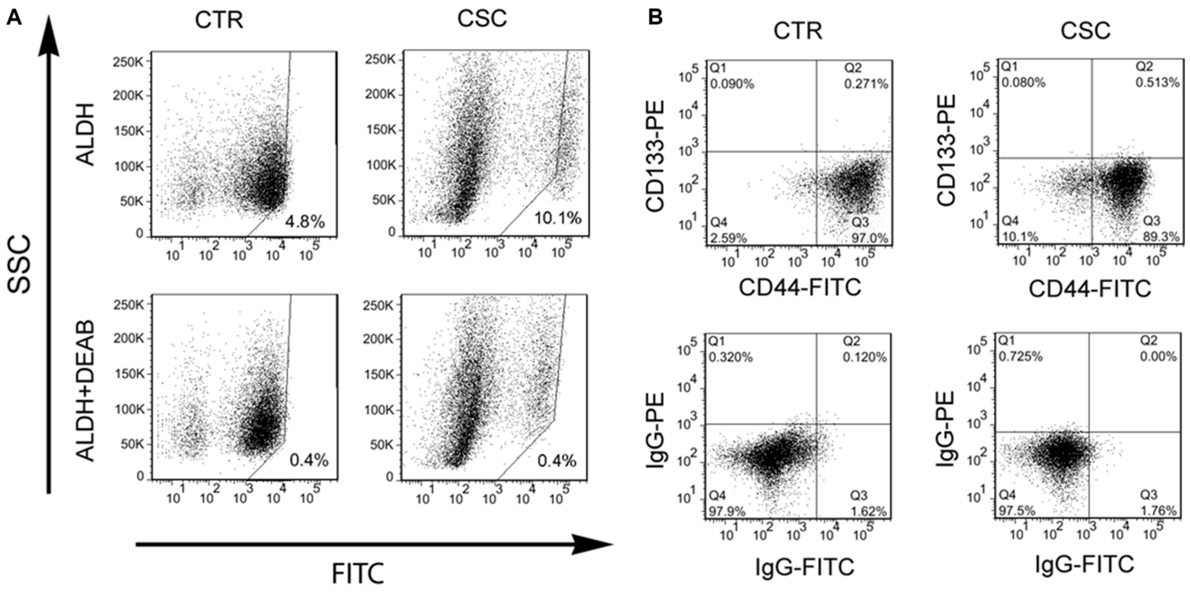

Supplementary Figure S1: Spheroid culture increased ALDH activity of A549 cells but did not increase the subpopulation of $CD133^+$/$CD44^+$ cells. (**A**) ALDH activity of A549 cells cultured under monolayer (CTR) or spheroid (CSC) conditions. (**B**) Flow cytometry of CD133 and CD44 double staining.

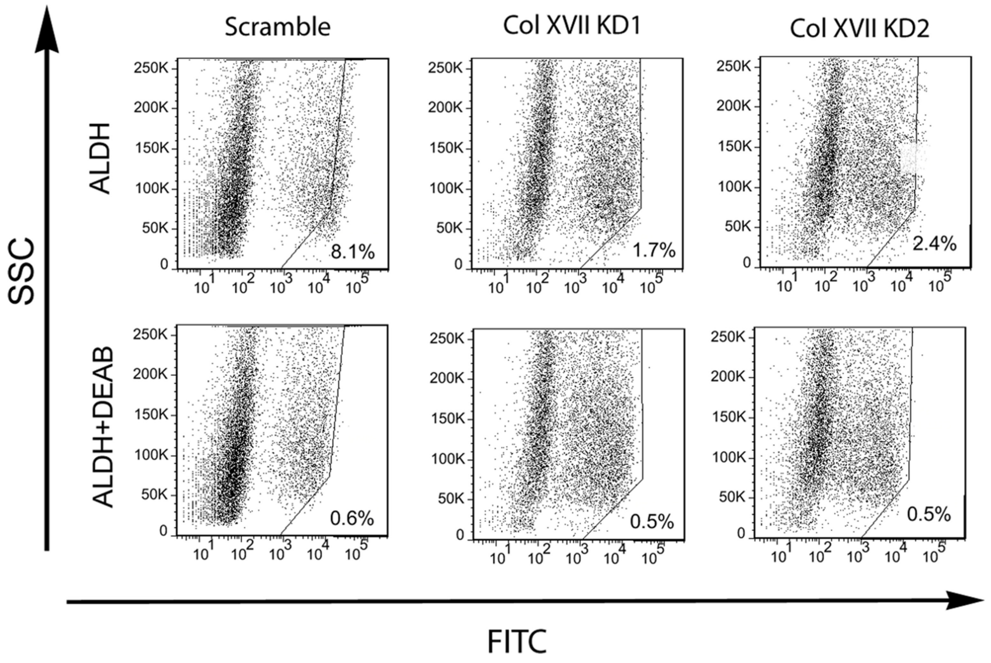

Supplementary Figure S2: Col XVII knockdown suppressed ALDH activity in spheroid culture. ALDH activity of A549 cells with Col XVII knockdown (KD) cultured under spheroid culture conditions.

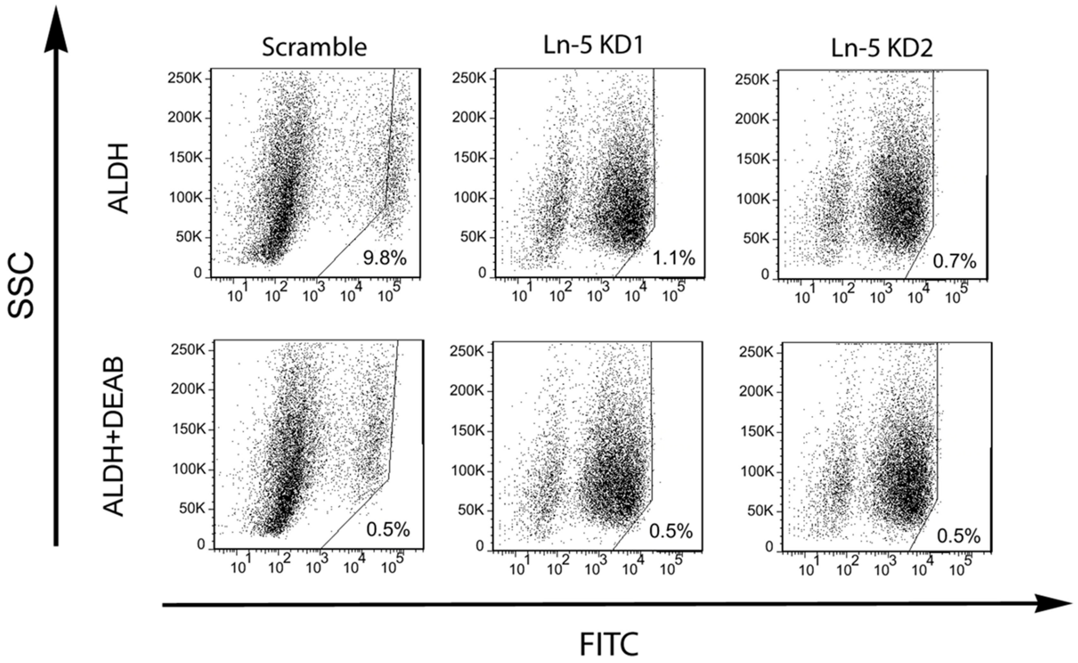

Supplementary Figure S3: Laminin-5 knockdown suppressed ALDH activity in spheroid cultures. ALDH activity of A549 cells with laminin-5 (Ln-5) knockdown (KD) cultured under spheroid conditions.

Supplementary Table S1: The results of microarray analysis in lung cancer cells cultured with or without spheroid medium

Gene Symbol	Entrez Gene	Fold change (Daoy-A549 vs. A549)	Gene Symbol	Entrez Gene	Fold change (Daoy-A549 vs. A549)	Gene Symbol	Entrez Gene	Fold change (Daoy-A549 vs. A549)
EPHB3	2049	3.801267	SELPLG	6404	4.317229	RAPH1	65059	3.9932764
LGALS3BP	3959	3.2198184	ITGAX	3687	5.878187	ITGB8	3696	14.027397
GPNMB	10457	4.010161	PCDHA1	9752	3.1180418	ITGA1	3672	7.540718
CYP1B1	1545	3.1746707	EDA	1896	4.386034	MPZL3	196264	4.4793787
CLDN7	1366	3.4990747	EDA	1896	4.386034	LSAMP	4045	4.3488445
ITGB2	3689	4.004871	CD84	8832	5.7293506	FREM1	158326	3.1782727
AGT	183	3.4379804	COL13A1	1305	6.530901	CD36	948	4.027919
CD93	22918	4.9117465	RET	5979	9.285199	LSAMP	4045	4.3488445
S100A8	6279	7.5469093	VCAN	1462	3.1193922	LMLN	89782	8.194991
SOX9	6662	5.083279	CADM3	57863	4.9649873	NCAM1	4684	7.178024
SOX9	6662	5.083279	TRO	7216	7.238525	GLDN	342035	9.6214905
NELL2	4753	12.269872	FN1	2335	3.4063003	F5	2153	26.5977
TPBG	7162	3.006093	PCDHGA9	56107	3.6755307	TINAG	27283	7.225211
S100A9	6280	12.334759	CEACAM1	634	3.2269318	PCDHGC4	56098	7.2429643
DPP4	1803	11.478296	CEACAM1	634	3.2269318	KIAA1462	57608	12.404792
ARHGEF17	9828	4.789358	COL6A1	1291	10.630096	SRCIN1	80725	3.2516563
COL7A1	1294	3.3296413	FN1	2335	3.4063003	COL12A1	1303	8.188657
COL16A1	1307	3.6154962	NCAM1	4684	7.178024	CADM2	253559	5.776657
SEBOX	7448	3.326291	PLXNC1	10154	6.370429	RASEF	158158	5.559342
CD22	933	5.7259383	MUC5B	727897	19.264805	FAT3	120114	4.915558
EPHB3	2049	3.801267	SNED1	25992	5.985906	NTN1	9423	9.707547
COL17A1	1308	3.4681318	SPON1	10418	3.2258537	HEPACAM	220296	16.221796
S1PR1	1901	3.434874	CLDN18	51208	4.3543906	TPBG	7162	3.006093
F5	2153	26.5977	ITGB4	3691	4.8426313	COL28A1	340267	5.9832883
F5	2153	26.5977	MUC5AC	4586	154.62657	HMCN2	256158	3.957205
DSC2	1824	7.3692884	LOC101059911	4586	8.943722	RASEF	158158	5.559342
PDPN	10630	5.857203	FN1	2335	3.4063003	RAPH1	65059	3.9932764
ADAM12	8038	4.166508	CDH6	1004	5.0363927	SIGLEC11	114132	11.466388
ITGB4	3691	4.8426313	LRRN2	10446	7.6544204	PELO	53918	3.3414896
CLDN10	9071	3.1693237	FN1	2335	3.4063003	RASEF	158158	5.559342
SRPX2	27286	3.9072075	COL14A1	7373	20.285978	ITGAL	3683	3.438435
CDH6	1004	5.0363927	COL4A3	1285	3.0568228	CNTNAP4	85445	4.5087533
DSG3	1830	6.2298107	ITGA2B	3674	6.1090703	CASS4	57091	3.252114
ANGPT1	284	3.2186298	COL7A1	1294	3.3296413	CDHR1	92211	3.9795825
PTPRD	5789	7.0242634	CD22	933	5.7259383	STAB1	23166	4.913923
HABP2	3026	5.636392	NR1D1	7067	3.0723758	ITGB2	3689	4.004871
CD36	948	4.027919	CDH8	1006	9.267938	FAT3	120114	4.915558
CDH16	1014	4.0000186	CNTNAP2	26047	6.279269	ITGAD	3681	5.08718
CEACAM1	634	3.2269318	SIRPG	55423	6.263203	SIGLEC16	400709	4.9326663
ITGA10	8515	14.7146845	PDPK1	5170	5.9578977	MAGI1	9223	3.7478273
EMR2	30817	3.11195	CD58	965	3.3299782	MAGI1	9223	3.7478273
CCR8	1237	7.0967956	MUC5B	727897	19.264805	CPXM2	119587	5.3738723
LOC101060681	7146	6.6257777	IL1B	3553	4.036926			
PECAM1	5175	31.220676	SORBS1	10580	12.195067			
LAMB3	3914	3.1289375	ITGA11	22801	3.3695698			
ENTPD1	953	3.9220545	PCDHA1	9752	3.1180418			
CEACAM1	634	3.2269318	CLDN2	9075	3.2165232			
SEPT5-GP1BB	100526833	7.1952767	GPR98	84059	6.1961865			
CDH17	1015	29.525864	TINAG	27283	7.225211			
SPP1	6696	3.4178011	RAPH1	65059	3.9932764			
